# Scalable global alignment for multiple biological networks

**DOI:** 10.1186/1471-2105-13-S3-S11

**Published:** 2012-03-21

**Authors:** Yu-Keng Shih, Srinivasan Parthasarathy

**Affiliations:** 1 Department of Computer Science and Engineering, Ohio State University, Columbus, OH, USA

## Abstract

**Background:**

Advances in high-throughput technology has led to an increased amount of available data on protein-protein interaction (PPI) data. Detecting and extracting functional modules that are common across multiple networks is an important step towards understanding the role of functional modules and how they have evolved across species. A global protein-protein interaction network alignment algorithm attempts to find such functional orthologs across multiple networks.

**Results:**

In this article, we propose a scalable global network alignment algorithm based on clustering methods and graph matching techniques in order to detect conserved interactions while simultaneously attempting to maximize the sequence similarity of nodes involved in the alignment. We present an algorithm for multiple alignments, in which several PPI networks are aligned. We empirically evaluated our algorithm on three real biological datasets with 6 different species and found that our approach offers a significant benefit both in terms of quality as well as speed over the current state-of-the-art algorithms.

**Conclusion:**

Computational experiments on the real datasets demonstrate that our multiple network alignment algorithm is a more efficient and effective algorithm than the state-of-the-art algorithm, IsoRankN. From a qualitative standpoint, our approach also offers a significant advantage over IsoRankN for the multiple network alignment problem.

## Background

Advances in technology have enabled scientists to determine, identify and validate pairwise protein interactions through a range of experimental methods such as two-hybrid analysis [1] and co-immunoprecipitation [2]. An important task, particularly when investigating networks of multiple species within a phylogeny tree, is that of *network alignment *where the objective is to understand which sub-networks are conserved across species in order to better understand the role of functional modules across the evolution of species [3-5]. Informally, given several (related) PPI networks and the protein sequence similarity scores between proteins within said networks, the goal of a network alignment algorithm is to find the best alignment, i.e., a mapping, which best represents functional orthologs among proteins within these networks. Additionally, this problem is also equivalent to identifying most biologically consistent match-sets, which are groups of proteins representing functional orthologs.

A PPI network can be represented as an undirected graph in which each vertex indicates a protein and each edge indicates an interaction between two proteins. The number of interactions is usually linear in the number of proteins in a PPI network. In other words, a protein only interacts with a limited portion of proteins in the same network. The graph is usually unweighted although an edge can often be associated with a confidence value indicating the probability that this edge is a true positive [6,7].

A local network alignment (LNA) algorithm aims to find highly similar pairs of motifs, i.e., subnetworks, across networks. The main drawback of LNA is that it might map one motif to several similar motifs [8-10]. Although this may sometimes reflect the gene duplication or gene fusion, LNA usually provides an unreasonable number of matches for a single protein. Moreover, it often suffers from a lack of a comprehensive picture of the network - a LNA usually aligns a very small portion of the entire network as most of the proteins do not appear in the alignment. Finally, a local alignment need not be consistent, i.e., a protein might be matched to more than two proteins where these matched proteins are not completely matched to each other. For this reason, global network alignment (GNA), which maps most of proteins across networks in a consistent and comprehensive way, has been proposed [8,11-13].

GNA requires that each protein in the network should be either matched to some proteins in other networks or marked as unaligned protein by the alignment, where the matches should be consistent [9]. Although a GNA can be estimated by using the result of a LNA as possible matches, the inconsistent matches of LNA typically limit the quality of the result alignment.

The main goals of GNA are to conserve the network topology and to ensure that the matched proteins' sequences are as similar as possible [7,8]. Since it involves a trade-off between these two competing goals, a GNA algorithm often employs a self-tuning mechanism to control this trade-off. A GNA algorithm may be a pairwise approach (where two networks are aligned) or a multi-way approach (where multiple networks are simultaneously aligned). A pairwise alignment is a special case of a multiple alignment. Multiple alignment strategies are often more biologically comprehensive since they can represent the degree of gene duplication as well as gene fusion. However, existing algorithms are not scalable enough to efficiently process a number of large networks with different trade-offs. Hence, we propose a scalable multiple global network alignment algorithm in this paper.

We present a simple but scalable approach for global multiple network alignment to exploit the sparsity of the PPI networks. Since fully integrating the sequence similarity and network topology is time-consuming especially in multiple networks, we consider these two goals independently. Our approach relies on first preprocessing the similarity scores and clustering all proteins into groups based on their similarity. We then adopt a seed-expansion heuristic strategy [14] in order to exploit the sparsity of the network, where a seed match-set is a set of proteins with high similarity scores. The idea here is analogous to region growth strategies that have found favor within the image processing community [15]. Subsequently, we develop a simple merging criterion to enable the multiple alignment of PPI networks.

We present a detailed empirical study which illustrates the benefits of the proposed approach on three real datasets. In short, we find that the proposed approach significantly improves over the state-of-the-art IsoRankN algorithm [10] along the twin axes of quality and efficiency. Specifically, from a qualitative perspective we found that our method can find more functionally consistent and more comprehensive alignments across all datasets. From an efficiency standpoint, our method is 50x to 500x faster than IsoRankN when executing on these three real datasets.

### Related work

Several algorithms, including MaWISH [16], Græmlin [17], DOMAIN [18], PathBLAST [19,20], Network BLAST [3], and Network BLAST-M [14], have focused on LNA. In order to efficiently obtain the maximal similarity score as conserving the maximal number of edges, Network BLAST and Network BLAST-M exploit the sparsity of the PPI networks by adopting seed-expanding method. Since LNA has several drawbacks as mentioned above, recent research has focused on GNA.

PATH [11], GA [8], NATALIE [12], NetAlignBP, NetAlignMR [13], and PISwap [5] all focus on GNA and all of them only address the pairwise alignment problem. They formulate the pairwise alignment problem into an objective function which combines the two goals of GNA: maximize the average similarity score and the number of conserved edges. Li et al. [7] shows that optimization of this objective function is equivalent to a binary integer quadratic programming problem, in which each binary variable represents whether a pair of proteins is matched together. Since the integer quadratic programming problem is NP-hard, all of these pairwise alignment algorithms are approximate algorithms. GA and PATH are based on graph matching algorithms that iteratively update a permutation matrix representing the matches between vertices of two graphs. However, this iterative process is time-consuming especially for networks consisting of thousands of proteins. NATALIE derives integer linear programming formulations from the integer quadratic programming problem and applies several relaxation algorithms to solve the linear programming problem. Bayati et al. [13] propose NetAlignBP and NetAlignMP for the constrained network alignment problem, in which a set of legal matches is given. NetAlignBP and NetAlignMP are efficient for large networks, but if the legal matches are all pairs of proteins across two networks, as in the general (unconstrained) network alignment problem, they both suffer from a large space demand. PISwap first utilizes the Hungarian algorithm [21], which is used to find the global matches which maximize the similarity score, and then use a method similar to 2-0pt [22], which is a heuristic algorithm for the traveling salesman problem, to swap the matches generated by the Hungarian algorithm in order to conserve edges. All of these pairwise alignment algorithms can find a near-optimal solution, but they cannot be adopted to the multiple alignment problem in a scalable fashion.

Græmlin 2.0 [23], IsoRank [24] and IsoRankN [10] generate multiple global alignments. Græmlin 2.0 automatically learns the scoring function's parameters, which indicate the weight of each feature of an alignment, from a training data set and then locally optimizes the scoring function in order to generate a near-optimal alignment. Græmlin 2.0 therefore needs a set of known alignments and thus the quality of Græmlin is sensitive to the training data set. IsoRank and IsoRankN generate a score matrix to capture both sequence similarity and network topology for all pairs of proteins, and IsoRankN uses a spectral clustering method to improve IsoRank. However, IsoRank and IsoRankN both need to iteratively generate and update the huge score matrix and hence both methods are inefficient for multiple networks consisting more than ten thousand proteins.

## Methods

### Definition of multiple network alignment

Assume we have *k *PPI networks {*G*_1_, *G*_2_,..., *G_k_*}. Each PPI network is an unweighted undirected graph *G_i _*= (*V_i_*, *E_i_*), where $V_{i}=\left\{v_{1},...,v_{\left|V_{i}\right|}\right\}$ is a set of proteins and each edge (*v_x_*, *v_y_*) ∈ *E_i _*represents an interaction between two proteins *v_x _*and *v_y_*. Let $V={⋃}_{i=1}^{k}V_{i},\;E={⋃}_{i=1}^{k}E_{i}$ and $n=\left|V\right|$ be the total number of proteins. A network alignment Ŝ is a set of mutually disjoint match-sets $\left\{S_{1},\;S_{2},\;\ldots ,\;S_{\left|\hat{\text{s}}\right|}\right\}$. Each match-set is a subset of proteins, i.e., $S_{x}\subset V$ for $1\leq x\leq \;|\hat{\text{S}}|$ and $S_{x}\cap S_{y}=\emptyset$ iff *x *≠ *y*. Note that each match-set can consist of any number of proteins from each network and some proteins would not belong to any match-set. The main concept is that proteins in the same match-set are matched together. In other words, an alignment is similar to a partition of all proteins but some proteins might not belong to any match-set.

### Metrics for alignments

In order to identify functional orthologs across multiple networks, the goal of PPI network alignment algorithms generally is to find corresponding matches across all networks as these match-sets should contain similar proteins and conserve as many interactions as possible [7,8]. Therefore, we adopt *the average similarity score *and *the number of conserved edges *introduced in [7] as the metrics for a network alignment.

The sequence similarity score for two proteins *v_x _*and *v_y _*is denoted by *sim*(*v_x_*, *v_y _*). *sim*(*v_x_*, *v_y_*) is set to 0 if *v_x _*and *v_y _*are in the same network since we only care about the similarity between proteins from different networks in the alignment problem. If *v_x _*and *v_y _*are in different networks, *sim*(*v_x_*, *v_y_*) is computed by BLAST or Pfam (Pfam 25.0) [25]. A higher similarity score indicates that the two proteins' sequences are more similar. Furthermore, both BLAST and Pfam scores for most of pairs of proteins are 0. The details about BLAST and Pfam are described in the result section.

The average similarity score of an alignment $\hat{\text{S}}$ is defined as the weighted average similarity score of the corresponding match-sets:

$$\frac{\sum_{i=1}^{|\hat{\text{s|}}}\left(\left|S_{i}\right|\times \;sim\;\left(S_{i}\right)\right)}{\sum_{i=1}^{|\hat{\text{s|}}}|S_{i}|},$$

where *sim*(*S_i_*) is the average similarity score of the match-set *S_i_*:

$$sim\left(S_{i}\right)=\frac{\underset{v_{x},v_{y}\in S_{i}}{\sum }sim\left(v_{x},v_{y}\right)}{\left(\underset{2}{\left|S_{i}\right|}\right)}$$

Since the similarity score between two proteins from the same network is zero, a match-set that includes proteins across several networks instead of only two networks will be preferred.

Let *S*(*v_i_*) denote the match-set containing *v_i_*. An edge (*v_x_*, *v_y_*) ∈ *E_i _*is conserved by an alignment Ŝ if (1) there exists an edge $\left(v_{x^{\prime }},\;v_{y^{\prime }}\right)\in E_{j},\;i\neq j$, such that *v_x _*and *v_x' _*are aligned together as well as *v_y _*and *v*_*y*'_, i.e., *S*(*v_x_*) = *S*(*v*_*x*'_) and *S*(*v_y_*) = S(*v*_*y*'_), or (2) *v_x _*and *v_y _*are aligned together, i.e., *S*(*v_x_*) = *S*(*v_y_*). Thereby, we count the number of conserved edges for an alignment by examining whether each edge e∈E satisfies these conditions. Note that an alignment which has only one single match-set containing all proteins would conserve all edges, but, the similarity score of this alignment would simply be the average similarity score of all pairs of proteins and therefore is not a meaningful alignment. We further define the term *strictly conserved edge *as an edge that satisfies the first condition and *sim*(*v_x_*, *v*_*x*'_) > 0 and *sim*(*v_y_*, *v*_*y*'_) > 0.

### Algorithm overview

As we mentioned before, optimization of the objective function consisting of these two goals for the pairwise network alignment problem is NP-hard. For this reason, we propose a heuristic method which independently considers these two goals in sequence in order to find a multiple alignment in feasible time. The main idea here is that there exist several *seed match-sets *(or *seeds *for simplicity), in which the proteins are highly similar to each other. Therefore, our approach is to initially ignore the network topology and use just the sequence similarity when forming the seeds. Here, we adopt clustering algorithms to effectively find the highly similar protein groups and then to form the seeds within the groups. Subsequently, the seeds will be expanded and merged by taking into account the network topology to form resulting match-sets.

**Figure 1:**
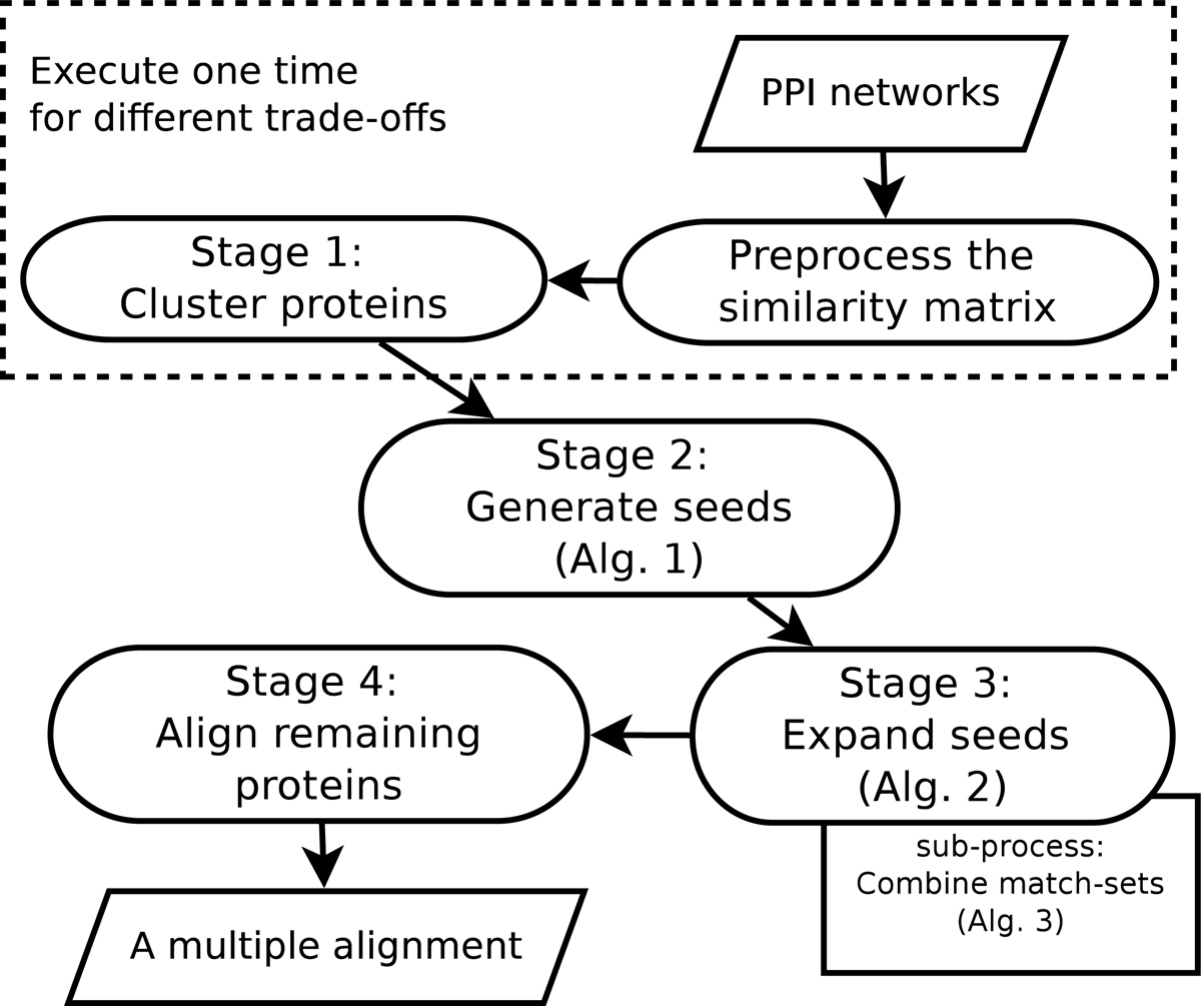
The procedure of our method.

The overall procedure of our algorithm is illustrated in Figure 1. As can be seen, our method first preprocesses the similarity matrix and then executes following four stages. Stage 1 applies a clustering method to cluster all proteins based on the preprocessed similarity matrix; Stage 2 generates seeds according to the clusters; Stage 3 expands the seeds to conserve edges, and finally, stage 4 aligns remaining proteins. The notations in the algorithms are explained in Table 1.

**Table 1:** Notations in the algorithms

*v.ali*	Whether the protein *v *is already aligned. The initial value is false.
*N *(*v*)	{*v*'**|***v*' is the neighbor of *v *and *v*'.*ali *= *false*}
*Net*(*v*)	The network where protein *v *is in.
*S*(*v*)	The match-set containing protein *v*.
*Net*(*S*(*v*))	The networks where at least one protein in *S*(*v*) is in.

### Preprocessing

The similarity matrix only represents the sequence similarity. If we only identify the seeds based on this similarity matrix, the seeds would not reflect any network topology. Therefore, we adopt a simple preprocessing method to integrate a part of network topology into the similarity matrix. Note that if we consider the whole network topology, the preprocessing would be too time-consuming (see [9]).

The main idea is that a pair of proteins with similar neighbors should be aligned together rather than a pair of proteins with a close similarity score but without any similar neighbors. Therefore we add an extra score which measures the similarity of two proteins' neighbors to the original similarity score. This extra score of a pair of proteins *v_a _*and *v_b_*, *Net*(*v_a_*) ≠ *Net*(*v_b_*), is initialized to 0 and this score is calculated in a greedy way: we select a pair of proteins $\left(v_{a}^{\prime },\;v_{b}^{\prime }\right)$ each time that (1) $v_{a}^{\prime }$ is *v_a_*'s neighbor; (2) $v_{b}^{\prime }$ is *v_b_*'s neighbor; (3) both of them have not been selected before; and (4) $\left(v_{a}^{\prime },\;v_{b}^{\prime }\right)$ should have the maximal similarity among all possible pairs satisfying the first three conditions. We add $sim\left(v_{a}^{\prime },\;v_{b}^{\prime }\right)$ to the extra score and then iteratively select the next pair until *v_a _*or *v_b_*'s neighbors are all selected, and the resulting extra score is eventually added to the original score. If the extra score is larger than the original score, we set the extra score to the same value of the original score, in order to avoid overemphasizing the neighbors' similarity.

**Figure 2:**
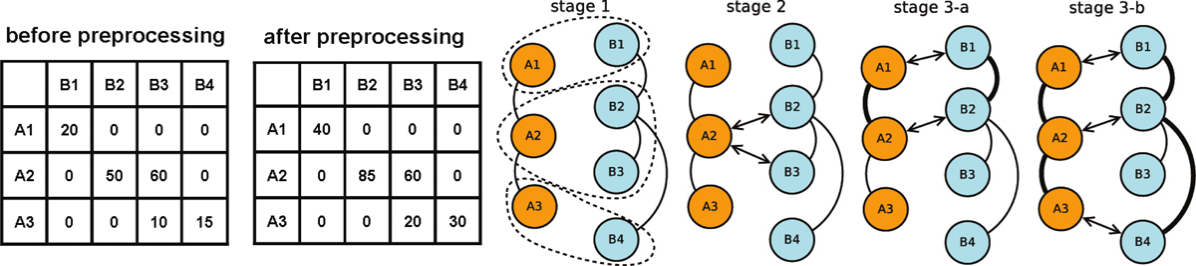
**An example with two networks, A and B.** The two tables are the similarity scores with and without preprocessing. The solid lines connecting two proteins in the same network are edges, and bold lines are conserved edges. The arrows across two networks are match-sets. The threshold *τ *is 50 here. Stage 1 shows the clustering result, which is {{A1, B1}, {A2, B2, B3}, {A3, B4}}. Stage 2 generates seed match-sets. Since here are only two networks, we do not merge any match-set and therefore the seed {A2, B3} is ignored. Stage 3 expands the alignment based on the seed {A2, B2}. There are no alignable pairs after stage 3, so stage 4 is not executed in this example.

An example is shown in Figure 2. *sim*(A2, B2) is initially 50. The extra score of (A2, B2) is equivalent to the sum of *sim*(A1, B1) and *sim*(A3, B4) However, the extra score of (A2, B3) is 0 since *sim*(A1, B2) and *sim*(A3, B2) are both 0. Therefore, the pair (A2, B2) is more favored than (A2, B3) as the match-set (A2, B2) can conserve more edges and obtain higher average similarity score by the subsequent stages than the match-set (A2, B3).

**Algorithm 1 **Seed Generation

**Input: **A set of clusters Č and the threshold of similarity *τ*.

**Output: **A set of seeds Ê.

1: Ê ← Ø;

2: **for all ***C *∈ Č **do**

3:    **for all ***v_x_*, *v_y _*∈ *C *: *Net*(*v_x_*) ≠ *Net*(*v_y_*) **do**

4:       **if ***sim*(*v_x_*, *v_y_*) ≥ *τ ***then**

5:          Ê ← Ê ∪(*v_x_*, *v_y_*);

### Seed generation via clustering

Seeds, which are pairs of proteins with high similarity, can be identified easily by pruning out all pairs of proteins with a similarity score lower than a threshold. However, this approach is not optimized since one protein might be similar to several proteins which are not mutually similar, and therefore the seeds generated by this approach might ruin the quality of match-sets formed by subsequent stages. As the seeds should be generated by globally considering their mutual similarity, we observe that this is equivalent to the clustering problem, in which mutually similar proteins should be clustered together.

We therefore adopt a clustering algorithm to identify the groups of similar proteins and then use Algorithm 1 to identify seeds. Algorithm 1 examines all possible pairs of proteins in each cluster (line 3), and then it uses the threshold parameter *τ *to determine whether a possible pair is similar enough to be a seed (line 4). Obviously, the lower the threshold is, the more seeds the algorithm generates. Hence, if the threshold is higher, we would generate more match-sets in subsequent stages. Since stage 3 considers network topology more, the resulting alignment should conserve more edges as the threshold becomes higher, but the average similarity score would be lower. If we generate too many clusters, each cluster will be too small, and therefore the resulting seeds cannot effectively span all networks. On the other hand, if there are too few clusters, the number of pairs of proteins we need to evaluate is very large, resulting in poor scalability. In this paper, we select $\frac{n}{k}$ as the number of clusters to achieve the balance between scalability and the span of match-sets, and we exclude proteins with zero similarity to all other proteins before clustering.

The clustering result directly affect the seeds generated by Algorithm 1, so it is very important for our algorithm to choose a clustering method which generates higher similarity score. Since the average similarity score of a match-set would be determined by the similarity scores of all pairs of proteins within a match-set, the clusters, which are used to generate seeds, should have nearly globular shape, i.e., any pair of proteins within a cluster should be reasonably similar. Density clustering methods, such as DBSCAN [26], are not suitable for our problem, since they tend to generate clusters with non-globular shape. Another characteristic here is that the number of clusters is $\frac{n}{k}$, and usual clustering methods cannot efficiently generate this huge number of clusters. K-medoids algorithm suffers from this problem; additionally, because K-medoids algorithm cannot effectively identify clusters with different sizes, a large group of similar proteins might be split by K-medoids algorithm. Hence, we choose hierarchical clustering methods with an approximated criterion function, which tend to generate clusters with globular shape and different sizes, for our algorithm. We consider the agglomerative method and the repeated-bisection method implemented by CLUTO (version: 2.1.2) [27]. We use two criterion functions, I1 and I2:

$$\text{I1}:\max\overset{k}{\underset{i=1}{\sum }}\frac{1}{n_{i}}\left(\underset{v,u\in S_{i}}{\sum }sim\left(v,\;u\right)\right)$$

$$\text{I2}:\max\overset{k}{\underset{i=1}{\sum }}\sqrt{\underset{v,u\in S_{i}}{\sum }sim\left(v,u\right)}.$$

**Algorithm 2 **Seed expansion on multiple networks

**Input: **A set of seeds Ê.

**Output: **A set of match-sets Ŝ

1: **for all **$v_{x}\in {⋃}_{i=1}^{k}V_{i}$**do**

2:    *S*(*v_x_*) ← {*v_x_*};

3: **for all **{*v_x_*, *v_y_*} ∈ Ê **do**

4:    Push ((*v_x_*, *v_y_*), *sim*(*v_x_*, *v_y_*)) in *pq*; //*pq *is a priority queue.

5: **while ***pq *is not empty **do**

6:    (*v_x_*, *v_y_*) ← *pq*.*pop*();

7:    Merge(*v_x_*, *v_y_*); //The boolean output of Algorithm 3 is ignored here.

8: **for all **match-set *S *: **|***S*| ≥ 2 **do**

9:    **for all **(*v_i_*, *v_j_*) ∈ *S *: *Net*(*v_i_*) ≠ *Net*(*v_j_*) **do**

10:       **for all **(*v_x_*, *v_y_*): *v_x _*∈ *N *(*v_i_*), *v_y _*∈ *N *(*v_j_*) **do**

11:          Push ((*v_x_*, *v_y_*), *sim*(*v_x_*, *v_y_*)) in *pq*;

12: **while ***pq *is not empty **do**

13:    (*v_i_*, *v_j_*) ← *pq*.*pop*();

14:    **if **Merge(*v_i_*, *v_j_*) **then**

15:       **for all **(*v**_x_***, *v**_y_***): *v_x _*∈ *N *(*v_i_*), *v_y _*∈ *N *(*v_j_*) **do**

16:          Push ((*v_x_*, *v_y_*), *sim*(*v_x_*, *v_y_*)) in *pq*;

17: Ŝ ← {match-set *S *: |*S*| ≥ 2};

### Seed-expansion strategy

We observe that if a new match-set which consists of the neighbors of an existing match-set is formed, two edges connecting the existing match-set to the new match-set are conserved. Therefore, we start conserving edges by first expanding the seeds, i.e., forming new match-sets consisting of the neighbors of seeds. Since we still want to obtain higher similarity scores during expansion, we adopt a priority queue which contains all expandable pairs of proteins in order to iteratively select the expandable pair with the highest similarity score. Once a new pair is popped from the priority queue and used to form a new match-set, we put the neighbors of the new match-sets into the priority queue in order to expand the alignment. Thereby, each time we pick a pair of proteins and align them together, we conserve two edges and expand the alignment. This method is very efficient to directly conserve a higher amount of edges as we still obtain high similarity scores.

**Algorithm 3 **Merge

**Input: **A pair of proteins (*v_i_*, *v_j_*) and three user-defined parameters *β*, *ρ*, and *ω*.

**Output: **A boolean value indicating whether the algorithm merges *S*(*v_i_*) and *S*(*v_j_*).

1: **if **|*S*(*v_i_*)| + |*S*(*v_j_*)| >ω _* _|*Net*(*S*(*v_i_*) ∪ *S*(*v_j _*))| **then**

2:    **return false**;

3: *maxSim ← max*{*sim*(*v_x_*, *v_y_*)|*v_x_*, *v_y _*∈ *S*(*v_i_*) ∪ *S*(*v_j _*)};

4: *count *= |{(*v_x_*, *v_y_*)|*v_x _*∈ *S*(*v_i_*), *v_y _*∈ *S*(*v_j_*), *sim*(*v_x_*, *v_y_*) >*maxSim *× *β*}|;

5: *expand Ratio *= |*Net*(*S*(*v_i_*) ∪ *S*(*v_j_*))|/*MAX*(|*Net*(*S*(*v_i_*))|, |*Net*(*S*(*v_j_*))|);

6: **if ***maxSim *> 0 **and **count _* _*expandRatio *≥ |*S*(*v_i_*)| × |*S*(*v_j_*)| × *ρ ***then**

7:    Merge *S*(*v_i_*) and *S*(*v_j_*);

8:    *v_i_*.*ali*, *v_j_*.*ali ***← true**;

9:    **return true**;

10: **else**

11:    **return false**;

Figure 2 shows an example, in which there is only one seed {A2, B2}. Among all 6 expandable pairs for that seed, {A1, B1} has the highest similarity score, so we first align A1 to B1 and conserve two edges (A1, A2) and (B1, B2). We then align A3 to B4, and conserve two edges (A2, A3) and (B2, B4). The resulting alignment is {{A1, B1}, {A2, B2}, {A3, B4}}, with 4 conserved edges and the average similarity score 85/3. Note that there are many possible alignments conserving four edges, but this alignment has the maximal average similarity score among them. It is interesting to note that although we can apply the Hungarian algorithm [21], which generates the alignment {{A1, B1}, {A2, B3}, {A3, B4}} that maximizes the average similarity score (95/3), for this pairwise alignment problem, this alignment does not conserve any edge and thus it is unlikely to be biologically meaningful. Furthermore, if we do not preprocess the similarity matrix, the seed is {A2, B3} and the resulting alignment is {{A2, B3}, {A3, B2}, {A1, B1}}, which only conserves two edges and its average similarity score, 80/3, is lower than the score of the alignment with preprocessing.

### Merging match-sets

In multiple alignments, each match-set usually consists of more than two proteins, some of which might be in the same network, so some of the seeds generated by Algorithm 1 and the match-sets formed by the seed-expansion strategy should be merged if the proteins in these match-sets are mutually similar enough. We introduce the procedure similar to agglomerative clustering in Algorithm 2 in order to merge match-sets. In agglomerative clustering, each individual object forms a cluster only containing itself at the beginning, and then two clusters are merged into one cluster each round. Here, each protein forms a match-set containing only itself at the beginning (line 1-2) and we iteratively merge two match-sets into one match-set according to the merging criterion. First, we merge those proteins contained by a seed to form a larger seed which might span more than two networks (line 3-7). Then, we apply the seed-expansion strategy, i.e., we expand the seeds through aligning the neighbors of the seeds together (line 8-16). Once a new pair of proteins is selected by the priority queue, we use the merging criterion to examine whether the two match-sets respectively containing these two proteins should be merged or not (line 14). If the merging criterion merges these two match-sets, we put all pairs of their unaligned neighbors in the priority queue in order to conserve more edges.

The merging criterion, Algorithm 3, determines whether two match-sets should be merged. If yes, the algorithm merges these two match-sets and then returns true. If no, it simply returns false. The first criterion is that the merged match-set cannot be larger than a size threshold, which is the number of networks this match-set crosses multiplied by the parameter *ω *(line 1-2). The suggested value of *ω *is [1.5, 2.5]. The intuition of this criterion is that a match-set should not be too large w.r.t. the number of species this match-set crosses; otherwise, the specific mapping of each protein in this match-set is ambiguous. If the merged match-set is equal to or smaller than the threshold, the second criterion is then applied: two match-sets will be merged if and only if *expandRatio *multiplied by the percentage of the similarity scores of all pairs across these two match-sets being larger than a threshold is larger than *ρ *(line 6). *expandRatio*, which is the expanding ratio of the number of species if these two match-sets are merged, is used to increase the possibility of two match-set crossing different networks being merged. The threshold is the product of the parameter *β *and the maximal similarity score among all pairs of proteins in the union of these two match-sets (line 4). The point of the second criterion is that if there are several non-highly similar pairs of proteins across two match-sets, we do not merge these two match-sets into a new match-set since the new match-set would tremendously decrease the average similarity score. The two parameters, *β *and *ρ*, in Algorithm 3 are both in the range (0, 1]. Generally, if *β *or *ρ *is larger, it is harder to satisfy the merging criterion, and thus the average size of the resulting match-sets becomes smaller and the match-sets in the alignment would cross less networks. Note that Algorithm 3 always merges the two match-sets containing only one protein. Additionally, if *ρ *is larger than 0.5 and there are only 2 networks, the match-sets are limited to contain 2 proteins, which is a usual constraint for pairwise alignments, so our algorithm can be applied to pairwise alignments.

### Aligning remaining proteins

Since there are no expandable pairs of proteins after stage 3 (Algorithm 2), we mainly focus on similarity scores in stage 4. The alignable pairs in this stage are all pairs of proteins for which (1) both proteins have not been aligned yet, and (2) the similarity score of the pair is not zero. The second condition is used to prune a large amount of pairs satisfying the first condition. We sort these alignable pairs and then iteratively pick the pair with the highest similarity score, and again we apply the merging criterion at the same time as Algorithm 3 to form match-sets across more than two networks. Note that since the PPI networks are usually well-connected, the number of remaining proteins for stage 4 is typically a very small portion of all proteins.

### Complexity analysis

Let *d_max _*be the maximal degree among all proteins. The preprocessing needs to calculate an extra score for each pair of proteins with a similarity score larger than 0. In order to calculate the extra score, we need to sort all possible pairs of neighbors, and therefore the time complexity is $O\left(d_{\text{max}}^{2}\log\left(d_{\text{max}}\right)\right)$. Let $n_{pair}^{\prime }$ be the number of pairs of proteins with non-zero similarity scores. The time complexity of the preprocessing step is $O\left(n_{pair}^{\prime }2d_{\text{max}}^{2}\log\left(d_{\text{max}}\right)\right)$ Note that, in practice for real data, the similarity matrix is typically a sparse matrix, where $n_{pair}^{\prime }\in \left[n,n\log n\right]$.

In stage 1, we adopt the I1 and I2 criterion functions in CLUTO, whose time complexity to cluster all proteins is *O*(*n*^2 ^log *n*). The complexity of stage 2, depends on the number of proteins in each cluster. For a cluster with *c *proteins, Algorithm 1 examines ${(}_{2}^{c})$ pairs of proteins. Since the number of clusters is set to $\frac{n}{k}$, in the worst case, there are $\frac{n}{k}-1$ clusters, each of which contains only one protein, and one cluster containing $n-\frac{n}{k}+1$ proteins, and therefore the time complexity of stage 2 is *O*(*n*^2^). However, if the clusters are balanced, i.e., each cluster consists of average *k *proteins, Algorithm 1 would only examine ${(}_{2}^{k})$ pairs of proteins for each cluster, resulting in *O*(*nk*^2^) time complexity.

Let *s_max _*be the maximal size of a match-set. Algorithm 3 needs to examine all pairs of proteins from two match-sets; therefore, its time complexity is $O\left(s_{max}^{2}\right)$. Algorithm 2 expands the alignment at most *n *times and each time it generates at most $\left(\underset{2}{s_{\max}}\right)d_{\text{max}}^{2}$ expandable pairs. Each expandable pair applies Algorithm 3 one time, and we use a priority queue to iteratively select one pair, so the time complexity of Algorithm 2 is $O\left(ns_{max}^{4}d_{\text{max}}^{2}\log\left(ns_{max}d_{\max}\right)\right)$. Since the number of interactions is usually linear or log-linear in the number of proteins in PPI networks, and we use parameters *ρ *and *β *to limit the match-sets merging, *d_max _*and *s_max _*are independent of the size of networks, and *s_max _*is only proportional to the number of networks *k*. Thereby, the time complexity of Algorithm 2 can be reduced to *O*(*k*^4^*n *log(*kn*)). Eventually, the worst case of stage 4 is to sort all pairs of remaining proteins, so its time complexity is *O*(*n'*^2 ^log *n*'), where *n*' is the number of unaligned proteins after stage 3. However, *n*' is usually a very small number for all proteins.

Hence, the total time complexity of our algorithm is *O*(*n*^2 ^log *n*), dominated by the clustering method. Moreover, if several alignments with different trade-offs are required, our method just needs to preprocess and execute the most time-consuming stage, clustering, once. All previous network alignment algorithms have to rerun the whole process from scratch [5,8,11-13].

## Result and discussion

### Experiment setup

In this section, we present experimental studies on real datasets. We performed our experiment on three real databases, DOMAIN [18], DIP (version 10/10/2010) [28] and BioGRID (version 3.0.65) [29]. We only extracted proteins with FASTA sequences in order to compute their similarity scores.

Table 2 presents a summary of these datasets. We use Gene Ontology terms [30] as standard functional annotations and exclude the annotation generated by electronic method (IEA). The similarity scores in DIP and BioGRID datasets are BLAST bit scores computed by BLAST package [31]. The raw BLAST bit scores are widely used to measure the similarity between two protein sequences in previous PPI alignment research [5,8-11,24]. In order to measure how similar two protein sequences are, we use the normalized BLAST bit scores given by [5] instead of the raw BLAST bit scores. The normalized BLAST bit scores are computed as

(1) $$\text{nBLAST}\left(v_{x},v_{y}\right)=\frac{\text{BLAST}\left(v_{x},v_{y}\right)}{\sqrt{\text{BLAST}\left(v_{x},v_{x}\right)}\times \sqrt{\text{BLAST}\left(v_{y},v_{y}\right)}}.$$

**Table 2:** Experimental datasets

Datasets	Species	# proteins	# PPIs	Percent of proteins with GO terms
DOMAIN	D. mela	5014	10884	95.8%
	S. cere	3481	11186	87.2%
	C. eleg	1864	2159	90.0%

DIP	D. mela	7486	22340	82.89%
	S. cere	5139	24821	93.87%
	H. sapi	5025	12705	95.22%
	C. eleg	3095	4891	68.27%
	E. coli	2953	11759	65.09%
	M. musc	1149	1171	97.39%
	H. pylo	708	1354	68.05%

BioGRID	D. mela	7210	24710	86.1%
	C. eleg	3420	6339	87.0%
	S. pomb	1995	12573	99.7%
	H. sapi	8282	45031	93.20%
	A. thal	1609	2861	94.59%

The raw BLAST bit scores favor long sequences while the normalized BLAST bit scores, whose values are between 0 and 1, are independent of the sequence length.

The DOMAIN dataset is a subset of an old-version dataset from DIP (version 10/14/2008). It requires that the sequence of each protein in the DOMAIN dataset should contain at least one domain, where a domain is defined as a FASTA sequence pattern. We then use the information of domains to calculate the similarity score: the similarity score in the DOMAIN dataset is

(2) $$sim\left(v_{x},\;v_{y}\right)=\underset{d\in D}{\sum }-\ln PV\left(v_{x},\;d\right)-\ln PV\left(v_{y},\;d\right),$$

where *D *is the set of common domains of *v_x _*and *v_y _*, and *PV *is the p-value calculated by Pfam [25,32]. Note that if two proteins do not have common domains, the similarity score is zero.

The experiments were performed on a dual core machine (Intel core i5 650) with 3.2 GHz of processor speed and 16GB of main memory. We discuss the tradeoff when tuning *τ *in the discussion of different clustering methods. The parameters (*β*, *ρ*, *ω*) in Algorithm 3 were set to default values (0.1, 0.5, 2.5) respectively, in all experiments unless otherwise noted.

In addition to the number of conserved edges and average similarity score, we used p-value [33], and the number of enriched GO terms [30] to evaluate functional consistency. The p-value is computed through the cumulative hypergeometric distribution. Let the total number of proteins of all networks be *N *with a total number of *M *proteins annotated with a certain GO term and a match-set containing *n *proteins in which *m *proteins are annotated with this GO term. The p-value of this match-set w.r.t this GO term is

$$\sum_{i=m}^{n}\frac{{(}_{i}^{M}){(}_{N-i}^{N-M})}{{(}_{n}^{N})}$$

and we select the lowest p-value among all GO terms as the p-value of this match-set. Following standard practice, a GO term is considered enriched if the p-value of one of the match-sets respect to this GO term is less than 10^-4^.

For multiple alignments, most existing algorithms do not align all proteins; in other words, some proteins would not belong to any match-set. *Coverage*, which is defined as the number of proteins contained by a match-set, can be used as a metric for evaluating multiple alignments. The wider the coverage is, the more comprehensive the alignment is for the networks. Since different alignments have different ranges of coverage, it is not objective to simply compare the number of conserved edges. Another metric that can be used here is *conserved edge rate *which is defined as number of conserved edges divided by the number of edges whose ending vertices are both covered by the alignment. Although coverage can be optimized by aligning every protein, it is difficult for an alignment with wider coverage to achieve high average similarity score and high conserved edge rate.

### Comparison between different clustering methods

**Figure 3:**
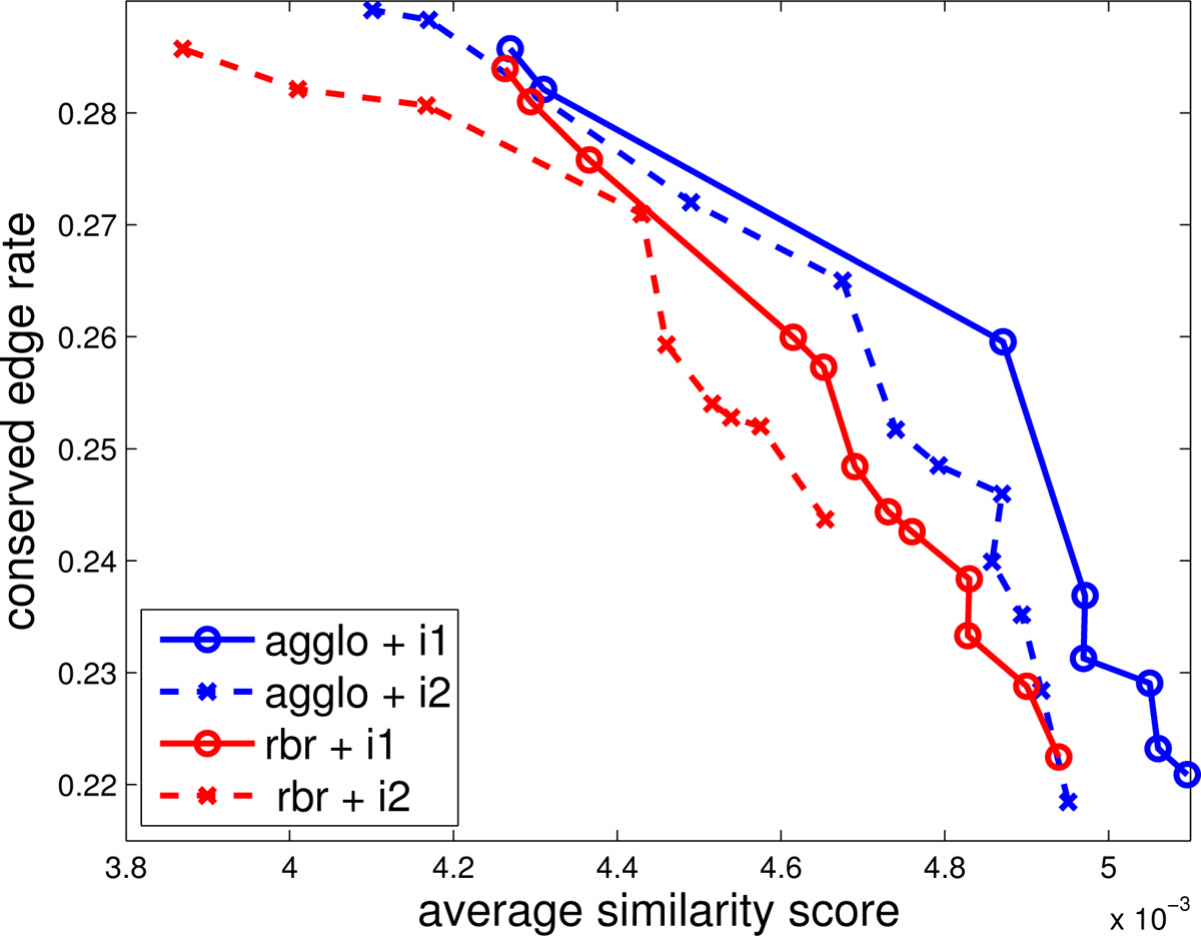
**The trade-offs in DIP dataset.** The average similarity score and the conserved edge rate of different clustering methods (agglo and rbr) and different criterion functions (i1 and i2).

Figure 3 shows the qualitative similarity scores and the conserved edge rates of the agglomerative method (agglo) and the repeated-bisection method (rbr) with criterion functions I1 and I2 in DIP dataset. In Figure 3, we show the effect of varying *τ*. We found that the range [30, 300] gives robust results. For the DIP and DOMAIN datasets, we found that *τ *∈ [0.1, 0.5] gives meaningful results. An obvious trend for all methods, is that the conserved edge rate decreases when greater weight is given to sequence similarity. Given a certain average similarity score, the agglomerative method always has higher conserved edge rate than the repeated-bisection method. Since the repeated-bisection method performs *m*-1 bisections to generate *m *clusters and each bisection just ensures the criterion function is locally optimized, the large number of clusters would adversely affects the global criterion function. In contrast, since the average number of proteins in each cluster is very small (the number of networks *k*), the agglomerative method is not only faster but also generates more similar clusters than the repeated-bisection method. Moreover, criterion function I1 can achieve larger highest similarity score than criterion function I2 in both the repeated-bisection method and the agglomerative method. Since I1 is similar to the measurement of the average similarity score of each cluster, clusters generated by I1 would result in match-sets with higher similarity scores. Similar results are observed in BioGRID and DOMAIN datasets. Hence, we adopt the agglomerative clustering method with criterion function I1 in the following section.

### Comparison with IsoRankN

When evaluating the performance, we compare our method with preprocessing against IsoRankN [10]. We empirically find that the match-sets detected by IsoRankN for different parameters is fairly stable. For each experiment, we pick the best of these. Table 3 reveals the comparison between our method and IsoRankN on DOMAIN, DIP, BioGRID datasets. Our method outperforms IsoRankN on these three data-sets in terms of coverage, average similarity score, and the number of enriched GO terms while the conserved edge rates of both algorithms are similar. The main reason is that the agglomerative clustering method in our algorithm can effectively identify groups of proteins with mutually similar sequences, while IsoRankN, which adopts spectral partitioning on its score matrix, cannot effectively find these groups. Moreover, the seed-expansion strategy can expand the alignment through the edges connecting to the seeds, and the stage 4 can align remaining proteins with high similarity, so the coverage is larger in our algorithm. It is important to note that since the number of interactions will increase (typically a multiple) with the number of covered proteins, it is difficult to retain the same conserved edge rate as the coverage is enlarged. We especially observe this for the network S. pombe in BioGRID dataset, which is denser than other networks. Overall we find that *our method conserves much more edges than IsoRankN, providing a more comprehensive topological mapping*.

**Table 3:** Comparison of quality between our method and IsoRankN

Datasets	DOMAIN		DIP		BioGRID	
**Algorithms**	**Ours**	**IsoRankN**	**Ours**	**IsoRankN**	**Ours**	**IsoRankN**

Coverage	8588	3372	24119	19555	21385	13928
Average similarity score	237.06	174.32	0.00509	0.00426	0.1735	0.0834
Conserved edges rate	.260 (6111 of 23507)	.160 (615 of 3138)	.2209 (17365 of 78611)	.086 (4696 of 54364)	0.1781 (13003 of 72975)	0.0685 (2586 of 37434)
# total enriched GO terms	1026	90	3871	1893	1123	523

The average similarity scores for DOMAIN dataset are computed by equation 2 and the average similarity scores for DIP and BioGRID datasets are normalized BLAST bit scores, computed by equation 1.

We also observe that the preprocessing can offer an improvement on the number of strictly conserved edges. The numbers of strictly conserved edges on DOMAIN, DIP, BioGRID datasets are 787, 1868, 1808 respectively without preprocessing while the numbers are 1125, 2243, 2216 respectively with preprocessing.

**Figure 4:**
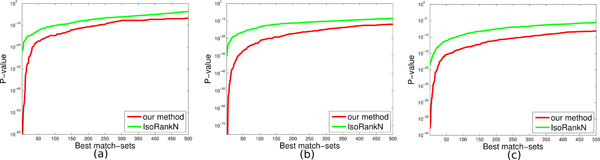
**The lowest 500 p-values on each dataset.** (a) DOMAIN (b) DIP (c) BioGRID.

Figure 4 shows the lowest (best) 500 p-values on these three datasets. Not surprisingly, both algorithms yield slightly lower p-values in DOMAIN than DIP, since all genes in DOMAIN have at least one sequence pattern so these genes tend to share similar functions. The p-values in three datasets clearly demonstrate that our method comfortably outperforms IsoRankN in terms of producing functionally relevant results. The main reason is that groups of proteins identified by the agglomerative clustering method can generate the seeds sharing highly similar functions. Subsequently, the seed-expansion method can form new match-sets sharing similar functions by conserving edges connecting to the seeds since two proteins interacting with each other tend to share the same functions. Finally, the merging criterion always preferentially merge two match-sets with a large number of mutually highly similar proteins, so the resulting match-sets potentially conserve biological functions.

**Figure 5:**
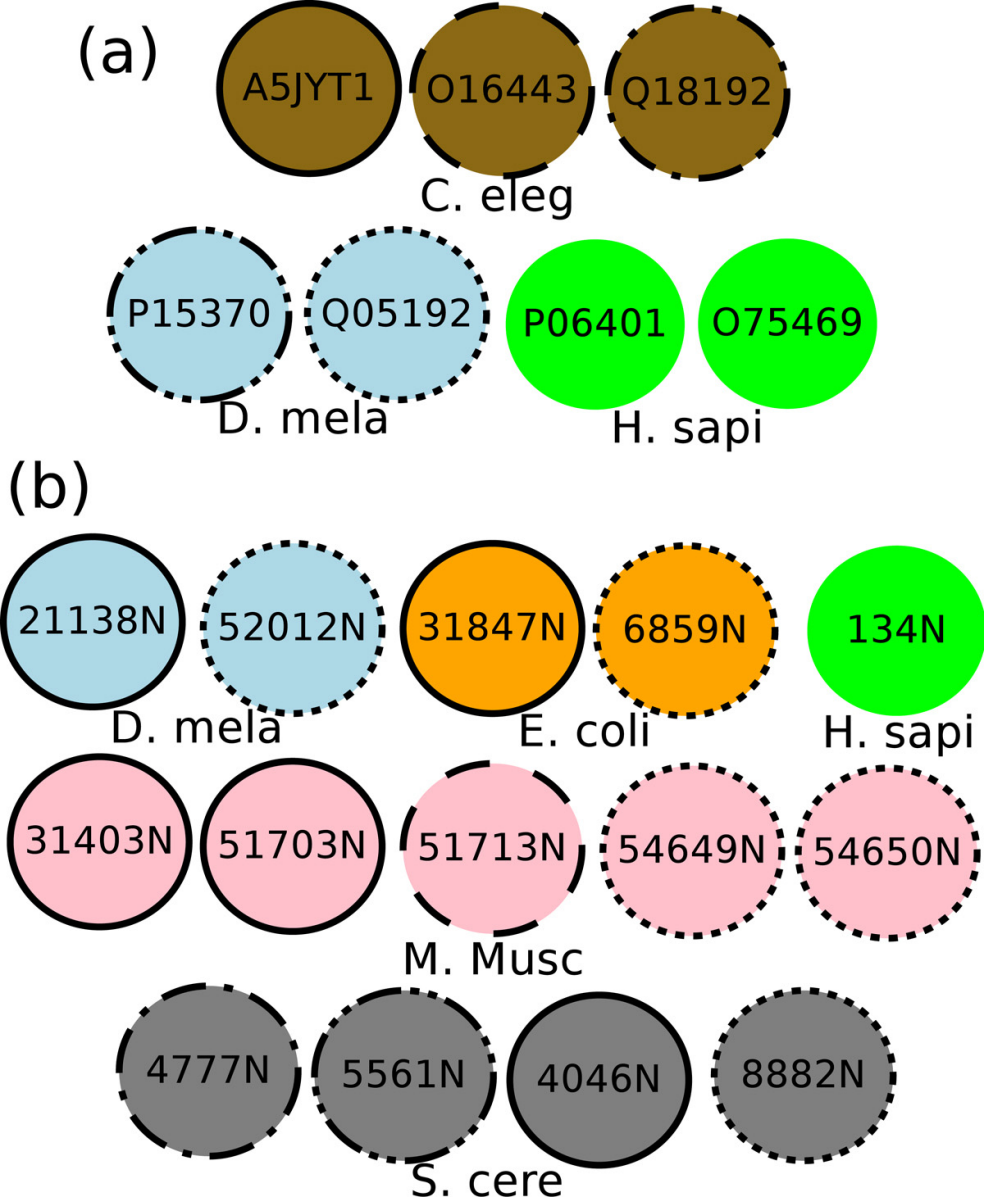
**The best match-set (with the lowest p-value) discovered by our algorithm.** Proteins with the same circle line style are in the same match-set formed by IsoRankN. Proteins without any circle are not covered by IsoRankN. The same color presents the same species. (a) DIP (DIP ID) (b) BioGRID (UniprotKB AC).

Figure 5 displays two of the top-ranked match-sets from our algorithm on DIP and BioGRID datasets respectively. For the DIP dataset, our method finds the best match-set consisting of 14 proteins from five networks, while IsoRankN does not cover one of them and aligns the remaining 13 proteins into five different match-sets. All 14 proteins have a subsequence highly similar to the domains PF03952.10 (*Enolase_N*) and PF00113.1 (*Enolase_C*) and except DIP-52012N and DIP-6859N, these proteins are all annotated with *glycolysis *(GO: 0006096). For the BioGRID dataset, our method discovers the match-set consisting of 7 proteins from three networks, but IsoRankN does not cover two of them and aligns the remaining five proteins in all different match-sets. These seven proteins are all associated with the domain PF00105.12 and six of them (except P15370) associated with the domain PF00104.24. Moreover, these seven proteins are all annotated with *steroid hormone receptor activity *(GO: 0003707). Our point here is that not only do we obtain greater coverage, we also report more functionally relevant groupings and do so at a fraction of the execution cost as we discuss next.

**Figure 6:**
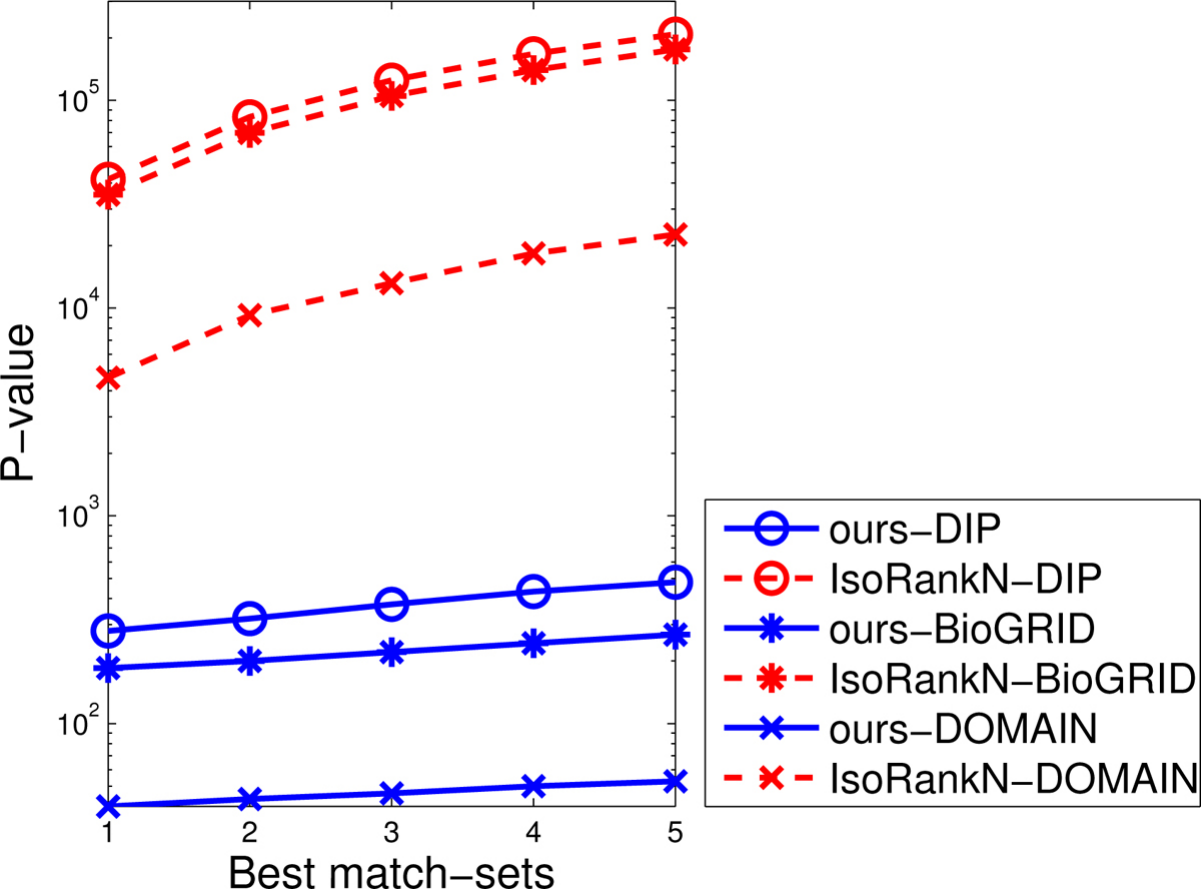
Execution time for generating several alignments.

Our method significantly outperforms IsoRankN in terms of execution time since IsoRankN iteratively updates a huge matrix whose size is proportional to the square of the total number of proteins in all networks. It is important to note here that the dominant cost in our method is the time to preprocess and run the clustering algorithm. These two steps only have to be executed once to generate alignments with different trade-offs. Keep in mind that IsoRankN has to be rerun from scratch if the trade-off weight is changed. For DOMAIN, DIP, and BioGRID datasets, the alignment stage only takes 5 to 40 seconds while the preprocessing step and clustering stage totally cost 37, 234 and 175 seconds respectively for our method, and IsoRankN takes 4621, 41719, and 35224 seconds on average respectively. Because IsoRankN does need to to rerun the whole process every time the threshold parameters are changed, as shown in Figure 6, the running time of IsoRankN is proportional to the number of alignments it generates; however, our method, which just needs to re-execute the alignment stages, is about three orders of magnitude faster to execute when generating five alignments. Note that once we need more alignments to be generated, the gap of execution time between our method and IsoRankN becomes larger.

To summarize, we find that *our algorithm provides both a significant qualitative as well as quantitative (efficiency) benefit over the state-of-the-art IsoRankN algorithm*.

## Conclusions

In this article, we present an efficient global PPI network alignment algorithm. Although our approach can also be applied to pairwise alignments, it mainly addresses multiple alignments, which are more comprehensive. In order to efficiently identify functional orthologs across multiple networks, we propose the merging criterion and apply the seed-expansion strategy and clustering techniques to conserve interactions and find similar protein sequences. Results on a number of real datasets highlight the effectiveness, efficiency, and scalability of our algorithm when we compared with the state-of-the-art multiple alignment algorithm, IsoRankN. From a qualitative standpoint, our approach also offers a significant advantage over IsoRankN for the multiple alignment problem.

In this work we do not explicitly consider the case of weighted PPI networks. In many cases weights representing the confidence associated with a detected interaction may be available. As part of future work, we would like to investigate the GNA problem with weighted interaction representing the evidence of interaction existence. A promising direction here is to develop a mechanism to integrate the similarity score with the interaction confidence. Another possible strategy is to adopt state-of-the-art graph clustering algorithms, such as [33,34], first and then use the sequence similarity to align similar clusters together.

## Competing interests

The authors declare that they have no competing interests.

## Authors' contributions

Y-KS developed software, conducted experiments, and drafted the manuscript. SP suggested the initial idea which was subsequently refined by both authors, guided the development and analysis of the method, helped design the experiments and co-wrote the manuscript. Both authors read and approved the final manuscript.
